# FRAMING AGE AND IMMIGRATION: OLDER IMMIGRANT WOMEN AND WELL-BEING IN A TIME OF SOCIAL INTOLERANCE

**DOI:** 10.1093/geroni/igae098.4329

**Published:** 2024-12-31

**Authors:** Jasmin Tahmaseb

**Affiliations:** West Chester University, West Chester, Pennsylvania, United States

## Abstract

In today’s contemporary troubling social climate, discourse against immigration is on the rise. In the U.S. older immigrants make up 13 % of the older adult population. The media often portrays both older adults and immigrants in biased and stereotypical ways, such portrayal can have a negative impact on the well-being of older immigrants and increase late life isolation and loneliness. This presentation examines the impact of how immigration and age are framed during the 2024 political campaign and the ways that those messages influence the lives of older immigrant women. Life history interviews exploring the goals, concerns, and supports of 18 older immigrant women (70+) indicate that the current political discourse on immigration has a negative influence on their lives, particularly as they navigate late life challenges. Media messages addressing the intersection of immigration and age are often negative increasing psychological distress among the women interviewed. We present examples and discuss the results of a content analysis of 10 news articles appearing over a 2-month period in several prominent newspapers, an analysis that illustrates the discriminatory nature of the ways that content is framed. We discuss the concerns, stressors, and resilience of participants as they struggle to manage late life challenges in a time of social intolerance and discrimination. The lives of older immigrants are often marked by cultural and economic challenges. Discriminatory framing of messages relating to both aging and immigration can increase life-course cumulative disadvantage negatively impact the well-being of an already vulnerable and socially marginalized group.

